# Supplementary material for: Evolutionary dynamics of Tomato spotted wilt virus within and between alternate plant hosts and thrips
Source: Sci Rep. 2020 Sep 25;10:15797. doi: 10.1038/s41598-020-72691-3 (PMC7519039; doi:10.1038/s41598-020-72691-3)
Supplement: Supplementary file 1 — Supplementary Information 1. [file 41598_2020_72691_MOESM1_ESM.pdf]

Supplementary Information:

Evolutionary dynamics of *Tomato spotted wilt virus* within and between alternate plant hosts and thrips

Authors: Casey L. Ruark-Seward[1], Brian Bonville[1], George Kennedy[1], David A. Rasmussen[1,2]

[1] Department of Entomology and Plant Pathology, North Carolina State University, Raleigh NC, USA

[2] Bioinformatics Research Center, North Carolina State University, Raleigh NC, USA

Corresponding author:
David A. Rasmussen
drasmus@ncsu.edu
North Carolina State University
Ricks Hall 312
1 Lampe Drive
Raleigh, NC, 27607, USA

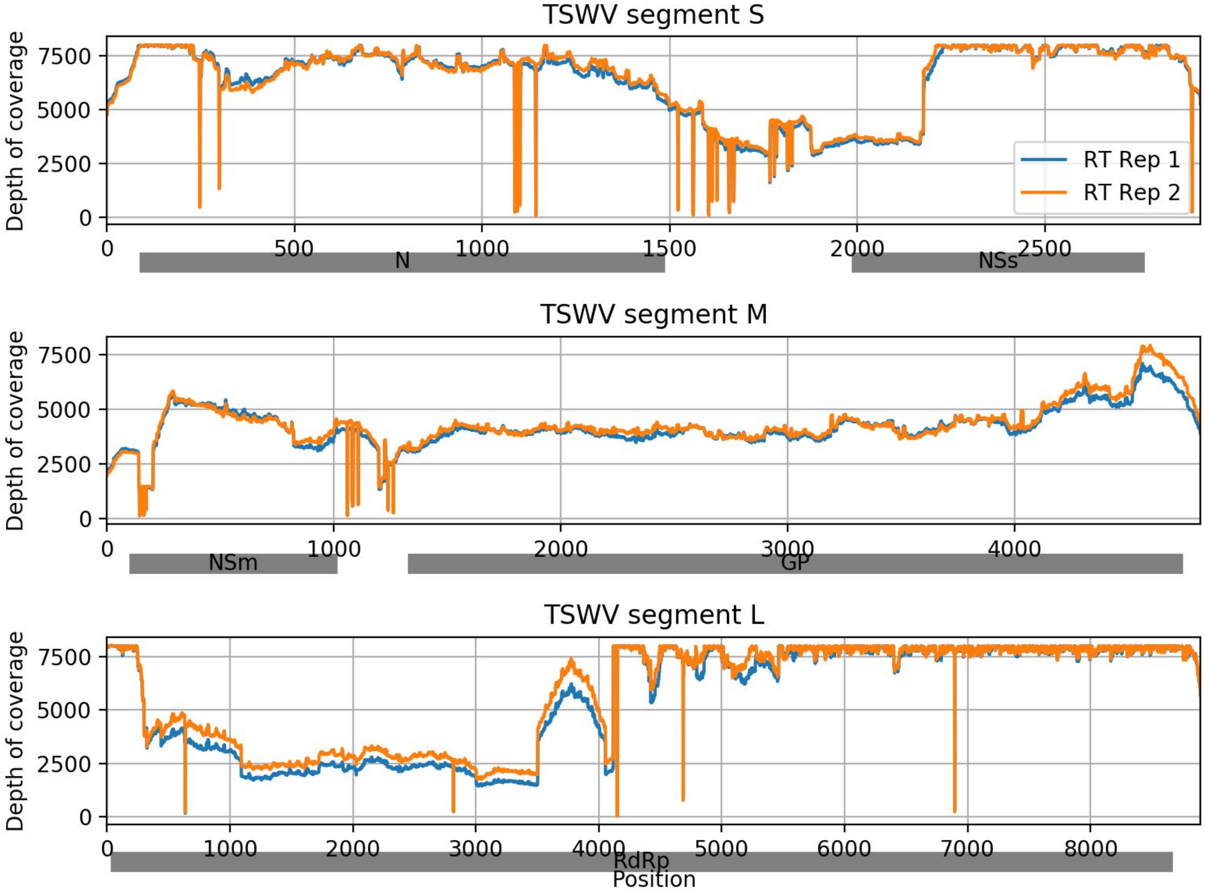

Supp. Figure 1: Sequencing coverage across the TSWV genome for the viral population at passage P0. Blue and orange lines show coverage obtained in paired sequence replicates obtained from independent reverse transcription reactions.

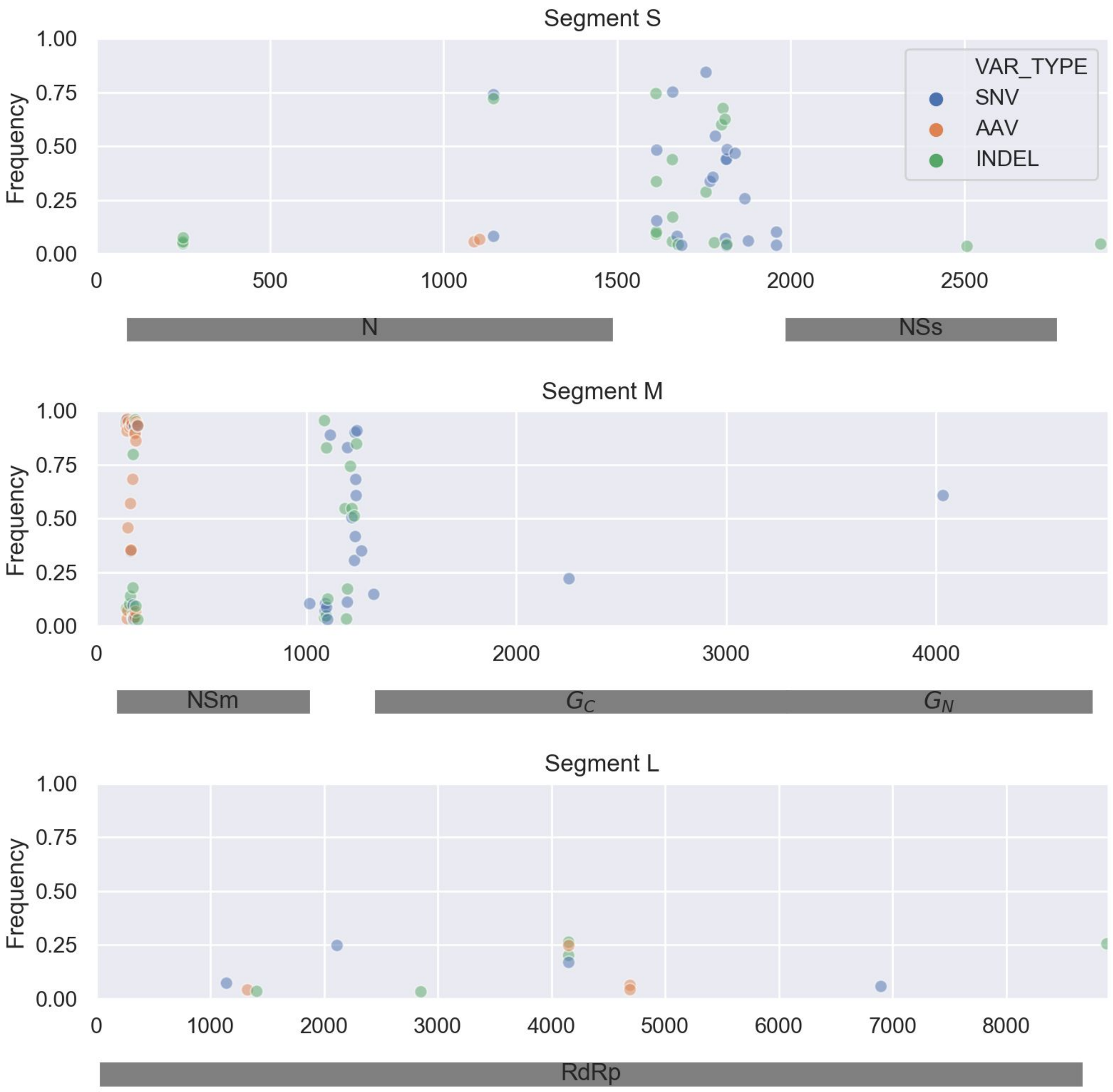

Supp. Figure 2: Viral genetic diversity in a naturally infected tomato leaf (TL2) collected in the field from the same plant as TF2. The frequency of each single nucleotide variant (SNV), amino acid variant (AAV) and indel is plotted at its respective position along the three segments of TSWV's genome. Shaded grey rectangles represent protein coding regions. Note: this is TL2_allVarFreqs_noLabels.png

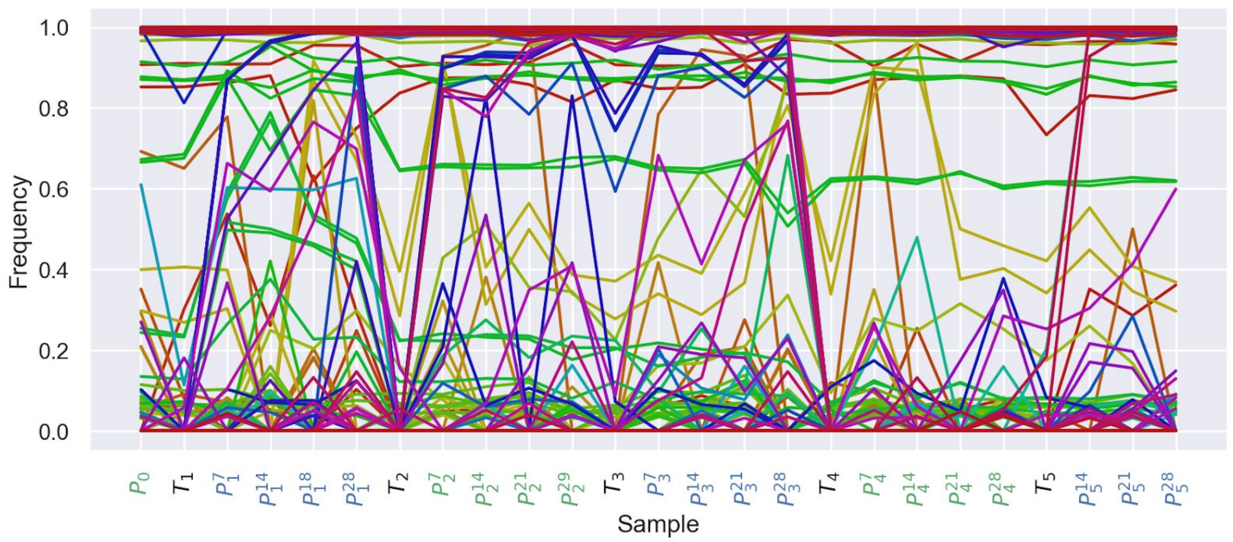

Supp. Figure 3: Time series showing the evolutionary dynamics of all single nucleotide variants through time in the *Alternating* Line. Sampling time points are colored by host; green = *Emilia*, black = thrips and blue = Datura.

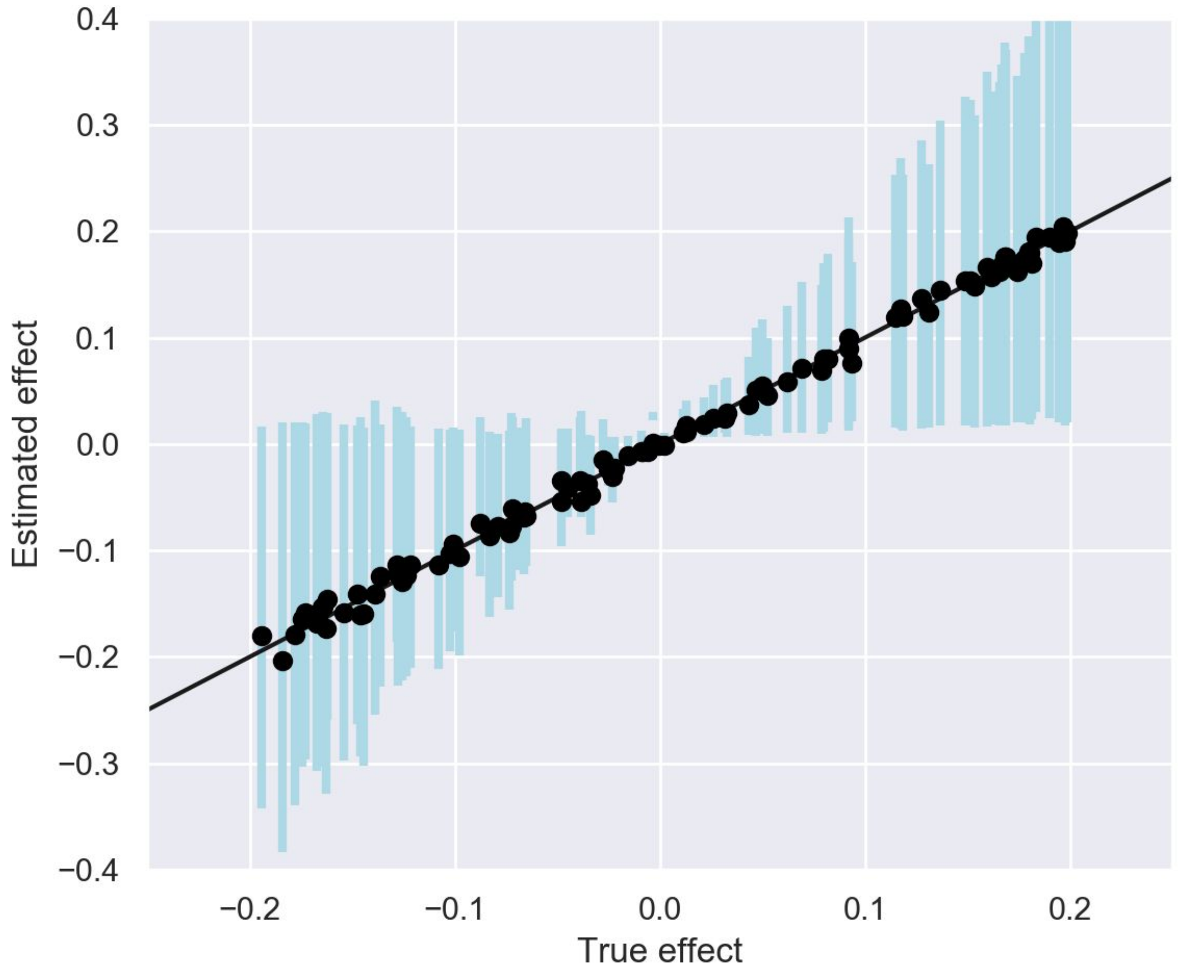

Supp. Figure 4: True versus estimated fitness effects inferred from 100 simulated time series. The posterior distribution of each estimated fitness parameter is summarized using the median (black dots) and 95% credible intervals (vertical blue lines). Note that the width of the credible intervals increases as the true fitness effects diverge from zero, reflecting greater uncertainty in our estimates. This increased uncertainty is expected under our model because larger fitness differences between variants will result in more unequal variant frequencies, which in turn increases the variance in simulated variant counts under the multinomial sampling model.

Supp. Table 1: Sequences of the TSWV L segment used to compute species level diversity.

RNA	Name of isolate	Region	Host	Size (nt)	Accession No.	Sample Date
S	CPNH9	Brazil	Tomato	2916	NC_002051	
S	Br20RB	Brazil	Pepper	2926	DQ915947	
S	Br20	Brazil	Pepper	2926	DQ915948	
S	KYN	China	Tomato	2970	JF960235	
S	KM-T	China	Tomato	2971	HQ402595	
S	CG-1	China	Lactuca sativa	2920	JN664252	
S	P105-1	Italy	Pepper	2927	DQ376177	
S	P105	Italy	Pepper	2927	DQ376178	
S	P166	Italy	Pepper	2925	DQ376179	
S	P267	Italy	Pepper	2927	DQ376180	
S	P272	Italy	Pepper	2934	DQ376181	
S	P105-43.14	Italy	Pepper	2927	DQ376182	
S	P105-44.7	Italy	Pepper	2926	DQ376183	
S	p170	Italy	Pepper	2954	DQ431237	
S	P170RB	Italy	Pepper	2930	DQ431238	
S	p105/2006RB	Italy	Pepper	2927	DQ915946	
S	p202/3RB	Italy	Pepper	2962	HQ830186	
S	p202/3WT	Italy	Pepper	2963	HQ830187	
S	p105-RB-MaxI	Italy	Pepper	2927	HQ839730	
S	p105-RB-MaxII	Italy	Pepper	2927	HQ839731	
S	ordinary strain	Japan	Unknown	2999	AB088385	
S	KD	Netherlands	Dahlia	2955	AF020660	
S	Unknown	South Korea	Unknown	2991	AB190819	
S	SPAIN-1	Spain	Tomato	2922	AY744479	
S	VE430	Spain	Pepper	2922	DQ376184	

S	VE427	Spain	Pepper	2922	DQ376185
S	K10	USA	peanut	3017	AF020659
S	CA-1	USA	Aster	2927	AY744468
S	CA-2	USA	Buttercup	2926	AY744469
S	CA-3	USA	Chrysanthe mum	2921	AY744470
S	CA-4	USA	Chrysanthe mum	2921	AY744471
S	CA-5	USA	Chrysanthe mum	2921	AY744472
S	CA-6	USA	Chrysanthe mum	2920	AY744473
S	CA-7	USA	Dahlia	2927	AY744474
S	CO	USA	Falso lulo	2923	AY744475
S	NC-1	USA	Dahlia	2959	AY744476
S	NC-2	USA	Peanut	3021	AY744477
S	NC-3	USA	Dahlia	2954	AY744478
S	M	USA	Emilia sonchifolia	3047	AY870391
S	T	USA	Emilia sonchifolia	3016	AY870392
S	K1 (Tomato NJ-JN)	South Korea	Tomato	2968	HM581936
S	K2 (Pepper1 CY-CN)	South Korea	Pepper	3013	HM581939
S	K3 (Pepper2 CY-CN)	South Korea	Pepper	3013	HM581942
S	K4	South Korea	Pepper	2971	KC261949
S	K5	South Korea	Stellaria aquatica	2975	KC261952
S	K6	South Korea	Stellaria media	2969	KC261955
S	K7	South Korea	Pepper	2967	KC261958
S	K8	South Korea	Lactuca indica	2977	KC261961

**S**	**K10**	**South Korea**	**Stellaria aquatica**	**2975**	**KC261964**
**S**	**K12**	**South Korea**	**Lettuce**	**2961**	**KC261967**
**S**	**K16**	**South Korea**	**Tomato**	**2973**	**KC261970**
**S**	**K17**	**South Korea**	**Stellaria media**	**2961**	**KC261973**
**S**	**K18**	**South Korea**	**Chrysanthe mum**	**3020**	**KC261976**
**M**	**D-191**	**Australia**	**Tomato**	**4824**	**HM015516**
**M**	**BR01**	**Brazil**	**Tomato**	**4821**	**S48091**
**M**	**BR01**	**Brazil**	**Tomato**	**4821**	**NC_002050**
**M**	**KYN**	**China**	**Tomato**	**4773**	**JF960236**
**M**	**CG-1**	**China**	**Lactuca sativa**	**4767**	**JN664253**
**M**	**p202/3RB**	**Italy**	**Pepper**	**4824**	**HQ830185**
**M**	**p202/3WT**	**Italy**	**Pepper**	**4824**	**HQ830188**
**M**	**Unknown**	**South Korea**	**Unknown**	**4768**	**AB190818**
**M**	**SPAIN-1**	**Spain**	**Tomato**	**4782**	**AY744492**
**M**	**SPAIN-2**	**Spain**	**Tomato**	**4785**	**AY744493**
**M**	**GRAU**	**Spain**	**Solanum lycopersicu m**	**4791**	**FM163370**
**M**	**GA-1L**	**Spain**	**Solanum lycopersicu m**	**4790**	**FM163371**
**M**	**ZO**	**Spain**	**Solanum lycopersicu m**	**4753**	**FM163372**
**M**	**LL-N.05**	**Spain**	**Solanum lycopersicu m**	**4752**	**FM163373**
**M**	**Ab1NL2**	**Spain**	**Solanum lycopersicu m**	**4784**	**HM015510**
**M**	**Cr1NL2**	**Spain**	**Solanum lycopersicu**	**4827**	**HM015511**

			m		
M	Da1NL2	Spain	Solanum lycopersicum	4830	HM015512
M	Gr1NL2	Spain	Solanum lycopersicum	4781	HM015513
M	Mon1NL2	Spain	Solanum lycopersicum	4785	HM015514
M	Ag1TL3	Spain	Solanum lycopersicum	4828	HM015515
M	Ber1TL3	Spain	Solanum lycopersicum	4825	HM015517
M	Llo2TL3	Spain	Solanum lycopersicum	4787	HM015518
M	Oller1TL3	Spain	Solanum lycopersicum	4825	HM015519
M	Pujol1TL3	Spain	Solanum lycopersicum	4825	HM015520
M	Sala1TL3	Spain	Solanum lycopersicum	4826	HM015521
M	ViTL3	Spain	Solanum lycopersicum	4787	HM015522
M	Gr5TL1	Spain	Solanum lycopersicum	4791	HM015523
M	Rib1TL1	Spain	Solanum lycopersicum	4786	HM015524
M	ALPA	Spain	Pepper	4782	HQ537114
M	D	USA	Unknown	4829	AF208497

M	Regular2A	USA	Unknown	4769	AF208498
M	CA-3	USA	Chrysanthe mum	4768	AY744481
M	NC-8	USA	Tomato	4774	AY744491
M	CA-4	USA	Chrysanthe mum	4767	AY744482
M	CA-5	USA	Chrysanthe mum	4767	AY744483
M	CA-6	USA	Chrysanthe mum	4764	AY744484
M	CA-7	USA	Dahlia	4766	AY744485
M	NC-3	USA	Dahlia	4827	AY744486
M	NC-4	USA	Tobacco	4773	AY744487
M	NC-5	USA	Tobacco	4787	AY744488
M	NC-6	USA	Pepper	4773	AY744489
M	NC-7	USA	Tobacco	4774	AY744490
M	T	USA	Emilia sonchifolia	4774	AY870389
M	M	USA	Emilia sonchifolia	4763	AY870390
M	K1 (Tomato NJ-JN)	South Korea	Tomato	4783	HM581935
M	K2 (Pepper1 CY-CN)	South Korea	Pepper	4768	HM581938
M	K3 (Pepper2 CY-CN)	South Korea	Pepper	4768	HM581941
M	K4	South Korea	Pepper	4781	KC261948
M	K5	South Korea	Stellaria aquatica	4792	KC261951
M	K6	South Korea	Stellaria media	4786	KC261954
M	K7	South Korea	Pepper	4785	KC261957
M	K8	South Korea	Lactuca indica	4787	KC261960
M	K10	South Korea	Stellaria	4791	KC261963

			aquatica			
M	K12	South Korea	Lettuce	4829	KC261966	
M	K16	South Korea	Tomato	4788	KC261969	
M	K17	South Korea	Stellaria media	4828	KC261972	
M	K18	South Korea	Chrysanthemum	4770	KC261975	
L	BR-01	Brazil	Tomato	8897	D10066	
L	BR-01	Brazil	Tomato	8897	NC_002052	
L	Hawaii	USA	Unknown	8640	AY070218	
L	KYN	China	Tomato	8910	JF960237	
L	K1 (Tomato NJ-JN)	South Korea	Tomato	8913	HM581934	
L	K2 (Pepper1 CY-CN)	South Korea	Pepper	8914	HM581937	
L	K3 (Pepper2 CY-CN)	South Korea	Pepper	8914	HM581940	
L	K4	South Korea	Pepper	8913	KC261947	
L	K5	South Korea	Stellaria aquatica	8913	KC261950	
L	K6	South Korea	Stellaria media	8913	KC261953	
L	K7	South Korea	Pepper	8913	KC261956	
L	K8	South Korea	Lactuca indica	8913	KC261959	
L	K10	South Korea	Stellaria aquatica	8913	KC261962	
L	K12	South Korea	Lettuce	8914	KC261965	
L	K16	South Korea	Tomato	8913	KC261968	
L	K17	South Korea	Stellaria media	8914	KC261971	
L	K18	South Korea	Chrysanthemum	8914	KC261974	
L	GC	South Korea	pepper	8925	MF159042	29-Jun-16

L	MJ	South Korea	pepper	8922	MF159048	9-Aug-16
L	JJ	South Korea	Capsicum annuum	8921	KY021437	13-Jun-16
L	PVR	Spain	pepper	8914	KP008132	29-Jun-05
L	Pujol1TL3	Spain	pepper	8914	KP008130	25-Jun-05
L	QLD1	Australia	Capsicum sp. cv. yolo wonder	8914	KT717691	2014
L	QLD2	Australia	Capsicum annuum cv. warlock		MG025802	20-Jul-15
L	LS3	South Korea	Leonurus sibiricus	8913	KM076651	14-Jul-13
L	YNrp	China	red pepper	8913	KM657120	1-Sep-13
L	YNta	China	tobacco	8913	KM657121	1-Sep-13
L	YNgp	China	green pepper	8913	KM657122	1-Sep-13
L	LL-N.05	Spain	tomato	8913	KP008128	2005
L	YN5574	China	Codonopsis pilosula	8913	MF422030	Jun-16
L	YN5575	China	dahlia	8913	MF422031	Jun-16
L	YN5577	China	Tropaeolu m majus	8913	MF422032	Jun-16
L	YN5573	China	pea	8913	MF590699	Jun-16
L	YN5576	China	Solanum lasiocarpu m	8913	MF590700	Jun-16
L	HLJ-1	China		8913	MG878873	Jul-17
L	LK-1	South Africa	tomato and Amaranthu s thunbergii		KY250488	15-Jun-15
L	PepCal_10	Italy	pepper	8909	MH763621	Jul-14

**L**	**PepCal_24**	**Italy**	**pepper**	**8909**	**MH763623**	**Jul-14**
**L**	**PepCal_12**	**Italy**	**pepper**	**8909**	**MK348941**	**Jul-14**
**L**	**LYE51**	**France**	**tomato**	**8674**	**MK792774**	**2005**
**L**	**Beijing-jh**	**China**	**chrysanthemum**	**8634**	**MK433644**	**Jun-18**

Green sequences are from Lian *et al.* (PLoS One, 2013). Other sequences are publicly available sequences downloaded from GenBank for this study.
